# The mixed Rossby–gravity wave on the spherical Earth

**DOI:** 10.1002/qj.3354

**Published:** 2018-09-28

**Authors:** Nathan Paldor, Itzhak Fouxon, Ofer Shamir, Chaim I. Garfinkel

**Affiliations:** ^1^ Fredy and Nadine Herrmann Institute of Earth Sciences, Edmond J. Safra Campus, Givat Ram, The Hebrew University of Jerusalem, Jerusalem, Israel

**Keywords:** mixed Rossby–gravity, shallow‐water waves, waves on a sphere, Yanai wave

## Abstract

This work revisits the theory of the mixed Rossby–gravity (MRG) wave on a sphere. Three analytic methods are employed in this study: (a) derivation of a simple ad hoc solution corresponding to the MRG wave that reproduces the solutions of Longuet‐Higgins and Matsuno in the limits of zero and infinite Lamb's parameter, respectively, while remaining accurate for moderate values of Lamb's parameter, (b) demonstration that westward‐propagating waves with phase speed equalling the negative of the gravity‐wave speed exist, unlike the equatorial β‐plane, where the zonal velocity associated with such waves is infinite, and (c) approximation of the governing second‐order system by Schrödinger eigenvalue equations, which show that the MRG wave corresponds to the branch of the ground‐state solutions that connects Rossby waves with zonally symmetric waves. The analytic conclusions are confirmed by comparing them with numerical solutions of the associated second‐order equation for zonally propagating waves of the shallow‐water equations. We find that the asymptotic solutions obtained by Longuet‐Higgins in the limit of infinite Lamb's parameter are not suitable for describing the MRG wave even when Lamb's parameter equals 10^4^. On the other hand, the dispersion relation obtained by Matsuno for the MRG wave on the equatorial β‐plane is accurate for values of Lamb's parameter as small as 16, even though the equatorial β‐plane formally provides an asymptotic limit of the equations on the sphere only in the limit of infinite Lamb's parameter.

## 1 INTRODUCTION

The existence of the mixed Rossby–gravity (MRG hereafter; also known as Yanai) wave was first predicted theoretically by Matsuno (1966) when he solved the linearized shallow‐water equations (LSWEs) on the equatorial *β*‐plane by deriving a time‐independent Schrödinger equation for the meridional velocity. The eigenfunctions and corresponding energy levels of this time‐independent Schrödinger equation yield the latitude‐dependent amplitudes and frequencies of the waves, respectively. In this description, the wave frequencies are obtained as the roots of a cubic in which one of the coefficients is the energy level of the Schrödinger equation. Thus, with the exception of the ground‐state level, each energy level yields three distinct frequencies corresponding to three distinct waves: eastward and westward inertia–gravity waves (EIG and WIG, respectively), and a westward‐propagating Rossby wave. At the ground‐state level, one of the three roots of the cubic equation, the one corresponding to *ω* = −*k* (where *ω* is the wave frequency and *k* the zonal wavenumber), leads to a singular zonal velocity component, and is thus discarded. The remaining two roots of the cubic equation for the ground‐state level correspond to the first EIG wave mode and westward‐propagating MRG wave. Oddly enough, the MRG wave on the equatorial *β*‐plane changes from a Rossby wave (at high *k*) to a WIG wave (at low *k*) right at the point where the *ω*(*k*) curve intersects the *ω* = −*k* line, even though *ω* = −*k* is a physically unacceptable solution and even though *k* is continuous in Cartesian coordinates.

**Figure 1:**
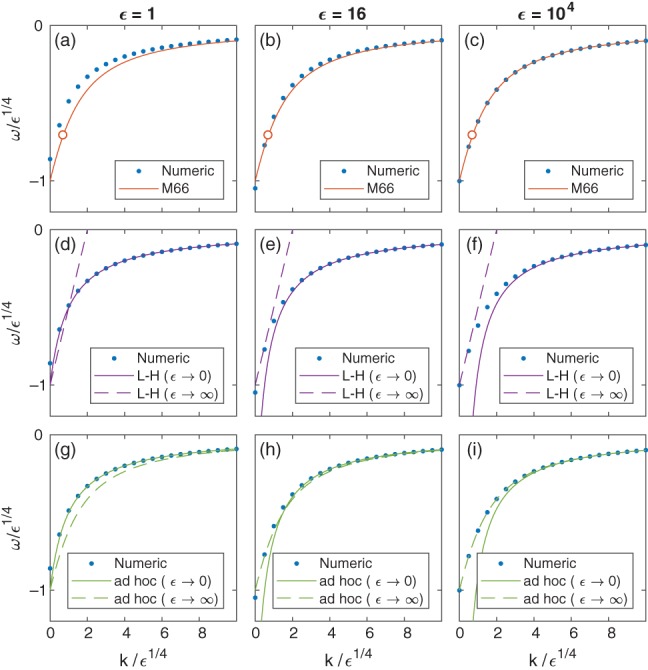
A comparison between the numerically computed (blue dots) dispersion relation ω(k) of the MRG wave mode and the analytic formulae suggested by Matsuno (1966) (red line, a–c), Longuet‐Higgins (1968) (purple lines, d–f). For comparison, we also show the expressions derived in Section 3 of the present study for the ad‐hoc solution (green lines, g–i). The ad‐hoc solution reproduces Matsuno's planar solution for ϵ→∞ (including the case ω = −k!) and L‐H's solution for ϵ→0. The numeric values were obtained using the shooting method as explained in Section 2.2. The comparison is shown for three values of ϵ from the range of values relevant to Earth: ϵ = 1 (a,d,g), ϵ = 16 (b,e,h) and ϵ = 10^4^ (c,f,i). In order to demonstrate their respective applicability, we show both of L‐H's formulae for ϵ→0 and ϵ→∞ in each panel regardless of the value of ϵ. Note, as indicated by the open circles at the intersection of the MRG wave mode and the ω = −k line of the top row panels, there exist no solutions with ω = −k on the equatorial β‐plane

A similar Schrödinger description of the LSWEs on a sphere was developed in De‐Leon and Paldor (2011) and Paldor *et al.*s*(2013). Recently, the authors of the present article used this approach in the study of eastward‐propagating waves, including the nondispersive Kelvin wave, on the sphere (Garfinkel *et al.*, 2017). In that study it was found that, in contrast to the equatorial *β*‐plane, where the Kelvin wave is a particular solution of the LSWEs that is not associated with an energy level of the Schrödinger eigenvalue equation, on a sphere the “Kelvin” wave should be classified as the lowest EIG wave mode derived from the ground‐state energy level of the Schrödinger eigenvalue problem. In addition, it was also shown in Garfinkel *et al.*s*(2017) that the LSWEs on the sphere reduce to those on the equatorial *β*‐plane only for *ϵ* ^1/4^ ≫ 1, where *ϵ* is Lamb's parameter (see definition in Section 2 below). However a comparison with numerical solutions on a sphere suggests that the dispersion relation obtained by Matsuno is valid even for small values of *ϵ*. Figure 1 demonstrates this last point by showing the numerical dispersion curve of the MRG wave obtained as described in Section 2.2 (blue dots), compared with the analytic expression obtained by Matsuno (red line in the top row) for three values of *ϵ* from the range of values relevant to Earth (see, for example, (Dunkerton, 1990)): *ϵ* = 1 (left column), *ϵ* = 16 (middle column), and *ϵ* = 10^4^ (right column). The new insight provided by the Schrödinger description in the study of the Kelvin wave on a sphere motivates a similar analysis for the MRG wave there. Moreover, the applicability of Matsuno's planar expressions at small values of *ϵ* on a sphere (though formally the planar theory is relevant to *ϵ* ^1/4^ ≫ 1) further motivates this detailed study of the MRG wave on the sphere, where the zonal velocity is regular, so all three roots of the ground‐state energy level describe physically acceptable (that is, regular) wave solutions of the LSWEs.

A more general approach to the study of the LSWEs on a sphere is described in the seminal work of Longuet‐Higgins (1968, L‐H, hereafter), who provides detailed numerical calculations of the solutions of the LSWEs on the sphere as well as asymptotic forms of the solutions in the limits *ϵ*→0 and *ϵ*→*∞*. However, while the MRG wave is clearly evident in his numerical calculations, Longuet‐Higgins provides no explicit discussion of this mode. Furthermore, the asymptotic forms obtained by Longuet‐Higgins are based solely on the value of *ϵ*, while ignoring its interplay with the wavenumber *k*. In particular, as noticed by Boyd (2018):

An important subtlety omitted from Longuet‐Higgins is that the degree of equatorial confinement of a mode, and therefore the accuracy of the equatorial beta plane in describing that particular Hough function, is not controlled by Lamb's parameter *ϵ* alone. Rather, if the longitudinal factor is exp(isλ) where *λ* is the longitude in radians and *s* is an integer, then latitudinal structure is proportional to $\exp(-\sqrt{\epsilon +s^{2}})$. Thus, it is possible for both Rossby waves and Kelvin waves to be equatorially trapped and therefore well approximated by the equatorial beta plane even when *ϵ* is zero, so long as the zonal wavenumber *s* is sufficiently large.

Indeed, as is shown in the middle row of Figure 1, even at *ϵ* = 10^4^, the asymptotic forms obtained by Longuet‐Higgins for *ϵ*→*∞* provide an accurate approximation of the dispersion relation only for small zonal wavenumbers (denoted here as *k*), while for most wavenumbers the asymptotic forms developed in Longuet‐Higgins for *ϵ*→0 provide a more accurate approximation.

This article is organized as follows. We start in Section 2 with a short description of the LSWEs on the sphere, the corresponding boundary‐value problems for zonally propagating wave solutions and the numerical method used here for solving them. In Section 3, we derive an ad hoc solution of the LSWEs on a sphere corresponding to the MRG wave that confirms its existence on the sphere. This ad hoc solution applies to any value of *ϵ* and reproduces the dispersion relations obtained by Longuet‐Higgins and Matsuno for *ϵ*→0 and *ϵ*→*∞*, respectively. In addition, the ad hoc solution provides a correction to the eigenfunctions in both limits and restores the $\sqrt{\epsilon +k^{2}}$ dependence mentioned by Boyd. However, like Matsuno's solution, the ad hoc solution of Section 3 is only valid for *ω* ≠ −*k*. Thus in Section 4 we provide a special treatment of the *ω* = −*k* case and show that on a sphere there exists exactly one solution with *ω* = −*k*. This solution fills in the missing point in the dispersion relation of the MRG wave mode on the equatorial *β*‐plane. In Section 5, we provide a complementary Schrödinger viewpoint on the MRG wave mode by classifying it as the ground‐state solution of the Schroödinger equations in the limits *ω* ^2^ ≪ *k* ^2^ and *k* = 0. The article ends in Section 6, where we discuss the results derived in the preceding sections.

## 2 GOVERNING EQUATIONS AND NUMERICAL METHOD OF SOLUTION

### 2.1 The linearized shallow‐water equations on the sphere

Let *a*, *g*, and Ω denote Earth's mean radius, gravitational acceleration, and angular frequency, and let *H* denote the mean thickness of the layer of fluid that covers the Earth. Using *a* and *H* as the horizontal and vertical length scales, respectively, and $\sqrt{a^{2}/gH}$ as the time scale (which implies that $\sqrt{gH}$ is the horizontal velocity scale), the nondimensional form of the LSWEs is

(1a) $$\begin{array}{ll} \frac{\partial u}{\partial t} & =\sqrt{\epsilon }v\sin\phi -\frac{1}{\cos\phi }\frac{\partial \eta }{\partial \lambda }, \end{array}$$

(1b) $$\begin{array}{ll} \frac{\partial v}{\partial t} & =-\sqrt{\epsilon }u\sin\phi -\frac{\partial \eta }{\partial \phi }, \end{array}$$

(1c) $$\begin{array}{ll} \frac{\partial \eta }{\partial t} & =-\frac{1}{\cos\phi }\left[\frac{\partial u}{\partial \lambda }+\frac{\partial }{\partial \phi }(v\cos\phi )\right], \end{array}$$

where 0 ≤ *λ* < 2*π* is the longitude and −*π*/2 ≤ *ϕ* ≤ *π*/2 is the latitude; *u* and *v* are the zonal and meridional velocity components; *η* is the free‐surface height anomaly (that is, deviation from the mean value of *H*); and *ϵ* = (2Ω*a*)^2^/*gH* is Lamb's parameter.

Seeking zonally propagating wave solutions with zonal wavenumber *k* (which is an integer) and frequency (or frequencies) *ω*, we let $\left\{u,v,\eta \right\}$ assume the form

(2) $$\left\{u,v,\eta \right\}=\left\{ũ(\phi ),-i\tilde{v}(\phi ),\tilde{\eta }(\phi )\right\}\exp[i(k\lambda -\omega t)].$$

The resulting boundary‐value problem for the unknown latitude‐dependent amplitudes $\left\{ũ(\phi ),\tilde{v}(\phi ),\tilde{\eta }(\phi )\right\}$ and corresponding frequency (frequencies) *ω* is

(3) $$\left[\begin{array}{ccc} 0 & \sqrt{\epsilon }\sin\phi & \frac{k}{\cos\phi }\\ \sqrt{\epsilon }\sin\phi & 0 & \frac{d}{d\phi }\\ \frac{k}{\cos\phi } & \left(\tan\phi -\frac{d}{d\phi }\right) & 0 \end{array}\right]\left[\begin{array}{c} ũ\\ \tilde{v}\\ \tilde{\eta } \end{array}\right]=\omega \left[\begin{array}{c} ũ\\ \tilde{v}\\ \tilde{\eta } \end{array}\right].$$

The −*i* factor in front of $\tilde{v}$ in Equation 2 is introduced for convenience, as it guarantees that Equation 3 and the subsequent formulae are all real.

Using the first row in Equation 3 to eliminate ũ from the second and third rows yields the following second‐order system for $\tilde{V}=\tilde{v}\cos\phi$ and $\tilde{\eta }$:

(4) $$\frac{d}{d\phi }\left[\begin{array}{c} \tilde{V}\\ \tilde{\eta } \end{array}\right]=\frac{1}{\omega \cos\phi }\left[\begin{array}{cc} k\sqrt{\epsilon }\sin\phi & k^{2}-\omega^{2}\cos^{2}\phi \\ \omega^{2}-\epsilon \sin^{2}\phi & -k\sqrt{\epsilon }\sin\phi \end{array}\right]\left[\begin{array}{c} \tilde{V}\\ \tilde{\eta } \end{array}\right],$$

which will be used in the subsequent analyses, along with the original Equation 3.

### 2.2 The shooting method

In order to substantiate the analytic results obtained in the following sections, we compare them with numerical solutions obtained using the shooting method for solving differential boundary‐value problems. For a general description of the method, the reader is referred to Press *et al.*s*(2007). Below, we describe the way this method is applied to calculate solutions of system Equation 4.

Since the diagonal elements of Equation 4 are odd functions of *ϕ* and the off‐diagonal elements are even functions of *ϕ*, it follows that one of the dependent variables is even and the other odd, so their product must vanish at the equator. Thus, we integrate Equation 4 from *ϕ* = *π*/2 − 10^−5^ to *ϕ* = 0 and search for values of the unknown frequency *ω* for which the “objective function”,

(5) $$F(\omega )=\tilde{V}\tilde{\eta }{|}_{\phi =0},$$

equals zero.

The integration is carried out using MATLAB's ode45 function, which implements an explicit fourth‐order Runge–Kutta method with adaptive step‐size based on the Dormand–Prince method (Dormand and Prince, 1980; Shampine and Reichelt, 1997).

In order to restrict the integration to the regular solution, we set $\tilde{V}(\pi /2-10^{-5})=1$ (which is a normalization of the linear differential system) and $\tilde{\eta }(\pi /2-10^{-5})=-(\omega +\sqrt{\epsilon })/k$ to ensure regularity at the singular poles (see (Garfinkel *et al.*, 2017)). Note the following. (a) A different time (and velocity) scale is used in Garfinkel *et al.*s*(2017). (b) The above relation is only valid for *k* ≠ 0, while for *k* = 0 the same regularity requirement yields $\tilde{\eta }(\pi /2-10^{-5})=2/\omega$, where again $\tilde{V}(\pi /2-10^{-5})=1$.

In order to locate candidate solutions, we inspect the graph of *F*(*ω*) visually and record the locations of its approximate roots (that is, values of *ω* where *F* changes sign). We then use these candidate roots as initial guesses in MATLAB's root‐finding function fsolve, which implements a trust‐region algorithm based on Powell's method (Powell, 1968; Press *et al.*, 2007). Once a root of the objective function is found, we integrate the equations again using that root and verify that the solutions and their derivatives are indeed continuous throughout.

## 3 AN AD HOC SOLUTION FOR THE MIXED ROSSBY–GRAVITY MODE

Our study of the MRG wave begins in this section with a derivation of an ad hoc solution of system Equation 4 that corresponds to the MRG wave and confirms its existence on the sphere for all values of *ϵ*.

**Figure 2:**
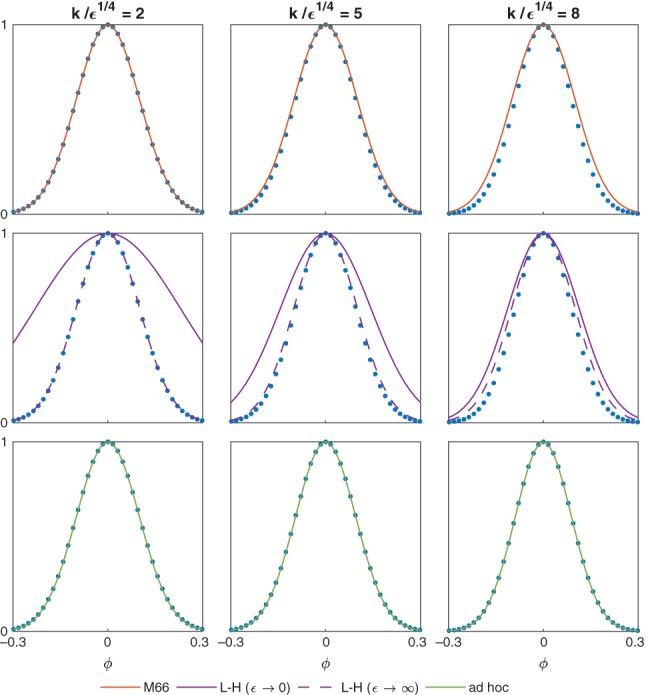
A comparison between the numerically computed (blue dots) meridional wind amplitude $\tilde{v}$ of the MRG wave mode and (a–c) the analytic formulae suggested by Matsuno (1966) (red line), (d–f) Longuet‐Higgins (1968) (purple lines), and (g–i) the ad hoc solution derived in Section 3 (green lines). The numeric values were obtained using the shooting method as explained in Section 2.2. The comparison is shown for a fixed value of ϵ = 10^4^ and three values of k, corresponding to (a,d,g) a small value of k/ϵ ^1/4^ = 2, (b,e,h) a moderate value of k/ϵ ^1/4^ = 5, and (c,f,i) a large value of k/ϵ ^1/4^ = 8. Note that the approximate solutions for ϵ→0 (solid, green) and ϵ→∞ (dashed, green) are indistinguishable from one another

**Figure 3:**
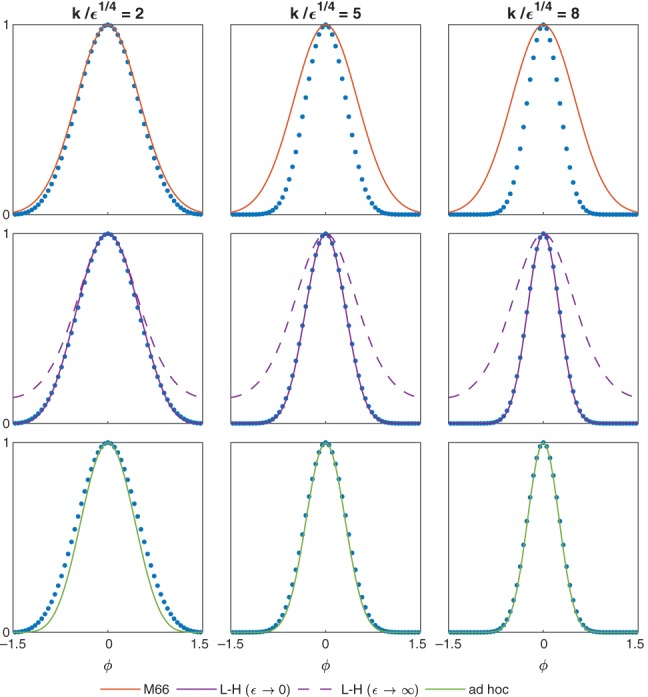
Same as Figure 2, but for ϵ = 16

We assume that $\tilde{V}(\phi )$ is of the form

(6) $$\tilde{V}=\cos^{\gamma }\phi ,$$

where *γ* is an unknown function of *ϵ* and *k* that will be determined shortly. This choice for the functional form of $\tilde{V}(\phi )$ is motivated by the envelope‐like shape found in our numerical solutions (shown below in Figures 2 and 3). Two notes are in order. (a) In Section 5, we show that, as is the case in Matsuno's solution and L‐H's *ϵ*→0 solution, this ad hoc solution fits the lowest mode (ground‐state) solution of the corresponding Schrödinger equation. Thus, the assumed functional form cuts off high‐order modes and keeps the lowest mode. (b) The normalization chosen here is $\max_{\phi }\{\tilde{V}\}=1$.

The corresponding structure of $\tilde{\eta }$, obtained by differentiating Equation 6 with respect to *ϕ* and substituting into the first row of Equation 4, is

(7) $$\tilde{\eta }=\frac{\gamma \omega +k\sqrt{\epsilon }}{\omega^{2}\cos^{2}\phi -k^{2}}\sin\phi \cos^{\gamma }\phi .$$

Similarly, differentiating Equation 7 with respect to *ϕ* and using the second row of Equation 4 yields, after division by $\cos^{\gamma -1}$,

$$\begin{array}{ll} & \frac{\left(\gamma \omega +k\sqrt{\epsilon })(\cos^{2}\phi -\gamma \sin^{2}\phi \right)}{\omega^{2}\cos^{2}\phi -k^{2}}\;\\ & +\frac{2\omega^{2}(\gamma \omega +k\sqrt{\epsilon })\sin^{2}\phi \cos^{2}\phi }{{\left(\omega^{2}\cos^{2}\phi -k^{2}\right)}^{2}}\;\\ & =\frac{\left(\omega^{2}-\epsilon \sin^{2}\phi \right)}{\omega }-\frac{k\sqrt{\epsilon }(\gamma \omega +k\sqrt{\epsilon })\sin^{2}\phi }{\omega (\omega^{2}\cos^{2}\phi -k^{2})}.\; \end{array}$$

Multiplying throughout by the common denominator $\omega {\left(\omega^{2}\cos^{2}\phi -k^{2}\right)}^{2}$, rewriting $\cos^{2}\phi$ in terms of $\sin^{2}\phi$, and collecting common powers of $\sin^{2}\phi$ yields

(8) $$\begin{array}{lcr} & & \omega \left(\omega \gamma +k\sqrt{\epsilon }-\omega \left(\omega^{2}-k^{2}\right)\right)\left(\omega^{2}-k^{2}\right)-\left(\omega \gamma +k\sqrt{\epsilon }\right)\\ & & \left[(\gamma +1)\omega \left(\omega^{2}-k^{2}\right)-\omega^{3}-k\sqrt{\epsilon }\left(\omega^{2}-k^{2}\right)\right]\sin^{2}\phi \\ & & +\omega^{2}\left(\omega \gamma +k\sqrt{\epsilon }\right)\left[(\gamma -1)\omega -k\sqrt{\epsilon }\right]\sin^{4}\phi \\ & & =-\left[2\omega^{4}+\epsilon \left(\omega^{2}-k^{2}\right)\right]\sin^{2}\phi \left(\omega^{2}-k^{2}\right)+\omega^{6}\sin^{4}\phi \\ & & \;+2\epsilon \omega^{2}\sin^{4}\phi \left(\omega^{2}-k^{2}\right)-\epsilon \omega^{4}\sin^{6}\phi . \end{array}$$

No approximations were made thus far, but in what follows we assume that the solution decays sufficiently fast with *ϕ* (that is, *γ* is large enough) that the $\sin^{4}\phi$ and $\sin^{6}\phi$ terms can be uniformly neglected compared with the $\sin^{0}\phi$ and $\sin^{2}\phi$ terms. The validity of this assumption will be confirmed a posteriori.

Equating the coefficients of the $\sin^{0}\phi$ and $\sin^{2}\phi$ terms yields the following two conditions:

(9) $$\left(\omega \gamma +k\sqrt{\epsilon }-\omega \left(\omega^{2}-k^{2}\right)\right)\left(\omega^{2}-k^{2}\right)=0$$

and

(10) $$\begin{array}{ll} & \left(\omega \gamma +k\sqrt{\epsilon }\right)\left[(\gamma +1)\omega \left(\omega^{2}-k^{2}\right)-\omega^{3}-k\sqrt{\epsilon }\left(\omega^{2}-k^{2}\right)\right]\;\\ & =\left[2\omega^{4}+\epsilon \left(\omega^{2}-k^{2}\right)\right]\left(\omega^{2}-k^{2}\right).\; \end{array}$$

The latter condition can be rewritten as a quadratic equation in *γ* as follows:

(11) $$\begin{array}{ll} & \omega^{2}\left(\omega^{2}-k^{2}\right)\gamma^{2}-\omega^{2}k^{2}\gamma \;\\ & -\left[2\omega^{4}+\epsilon \left(\omega^{2}-k^{2}\right)\right]\left(\omega^{2}-k^{2}\right)\;\\ & -k^{2}\sqrt{\epsilon }\left[k\omega +\sqrt{\epsilon }\left(\omega^{2}-k^{2}\right)\right]=0.\; \end{array}$$

We observe that, for *γ* ≠ *ϵ* ^1/2^, *ω* ^2^ = *k* ^2^ is not a solution of Equations 9 and 10. Thus, dividing Equation 9 through by (*ω* ^2^ − *k* ^2^) yields

(12) $$\frac{k\sqrt{\epsilon }}{\omega }-\omega^{2}+k^{2}=-\gamma .$$

Substituting *γ* in Equation 11, we obtain the dispersion relation,

(13) $$\begin{array}{lcr} & & \left(\omega^{2}-k^{2}\right){\left(k\sqrt{\epsilon }-\omega^{3}+k^{2}\omega \right)}^{2}+\omega k^{2}\left(k\sqrt{\epsilon }-\omega^{3}+k^{2}\omega \right)\\ & & -\left[2\omega^{4}+\epsilon \left(\omega^{2}-k^{2}\right)\right]\left(\omega^{2}-k^{2}\right)\\ & & -k^{2}\sqrt{\epsilon }\left[k\omega +\sqrt{\epsilon }\left(\omega^{2}-k^{2}\right)\right]=0, \end{array}$$

which can be written as

$$\begin{array}{lcr} & & \left(\omega^{2}-k^{2}\right){\left(k\sqrt{\epsilon }-\omega^{3}+k^{2}\omega \right)}^{2}-\omega^{2}k^{2}\left(\omega^{2}-k^{2}\right)\\ & & -\left[2\omega^{4}+\epsilon \left(\omega^{2}-k^{2}\right)\right]\left(\omega^{2}-k^{2}\right)-k^{2}\epsilon \left(\omega^{2}-k^{2}\right)=0. \end{array}$$

or

(14) $${\left(\frac{k\sqrt{\epsilon }}{\omega }-\omega^{2}+k^{2}\right)}^{2}=k^{2}+2\omega^{2}+\epsilon .$$

From Equation 12, it follows that

(15) $$\gamma =\sqrt{k^{2}+2\omega^{2}(k)+\epsilon }.$$

The last two equations are our main result, providing the dispersion relation and exponent *γ*, respectively. They hold irrespective of *ϵ*, provided the ad hoc solution is valid, that is, *γ* ≥ 1. In the limit of large *ϵ*, we have, from Equation 14,

(16) $$k^{2}+\sqrt{\epsilon }\frac{k}{\omega }-\omega^{2}\approx -\sqrt{\epsilon }.$$

This dispersion relation is identical to the dispersion relation of the lowest mode obtained by Matsuno (1966) (note that Matsuno's scaling is obtained from the present scaling by making the transformations *k*→*ϵ* ^1/4^ *k* and *ω*→*ϵ* ^1/4^ *ω*).

The only negative root of Equation 16 is (recall that *ω* ≠ −*k*)

(17) $$\omega =\frac{k}{2}-\sqrt{{\left(\frac{k}{2}\right)}^{2}+\sqrt{\epsilon }}.$$

Thus, in the limit of large *ϵ*,

(18) $$\gamma =\sqrt{2k^{2}+2\sqrt{\epsilon }-k\sqrt{k^{2}+4\sqrt{\epsilon }}+\epsilon }.$$

In the limit of small *ϵ*, the frequency scales as $\sqrt{\epsilon }$, so we find from (15) that *γ*≈*k*, which is the well‐known solution for the meridional structure derived from the Legendre polynomial expansion. An expression for the dispersion relation can be derived in this limit by setting $\omega =\sqrt{\epsilon }\tilde{\omega }$ in Equation 14, which, to leading (that is, zeroth) order, yields

(19) $${\left(\frac{k}{\tilde{\omega }}+k^{2}\right)}^{2}\approx k^{2}.$$

Rearranging, we find that

(20) $$\omega =-\frac{\sqrt{\epsilon }}{k+1}.$$

This dispersion relation is identical to the dispersion relation of the lowest mode in L‐H's equation (4.9). High‐order corrections to this leading‐order expression can be derived readily from the complete Equations 14 and 15. For instance, the next‐order correction for *γ* can be obtained by substituting the dispersion relation, Equation 20, in Equation 15.

The above expressions were derived by neglecting the $\sin^{4}\phi$ and $\sin^{6}\phi$ terms in Equation 8. In the limit of large *ϵ*, we can assume that $\sin^{2}\phi ∼1/\sqrt{\epsilon }$, since the solution decays fast with latitude. We find that $\sin^{6}\phi$ is of the order of $\sqrt{\epsilon }$ and the $\sin^{4}\phi$ term is of the order of *ϵ*. However, the zeroth‐order and second‐order terms in $\sin^{2}\phi$ are of order *ϵ* ^3/2^, confirming the validity of our solution at $\sqrt{\epsilon }\gg 1$. Similarly, at *ϵ* ≪ 1 we have in Equation 8 that $\sin^{6}\phi$ is of order *ϵ* ^3^ and the $\sin^{4}\phi$ term is of order *ϵ* ^2^. In contrast, the zeroth‐order and second‐order terms in $\sin^{2}\phi$ are of order *ϵ*, confirming the validity of our approximation and solution at *ϵ* ≪ 1. With these approximations Equations 6 and 7 for V and *η*, respectively, yield an explicit expression for u (see first row in Equation 3) that yields *u* = *η* for large *ϵ*.

It is readily seen from the above that $\gamma =\sqrt{k^{2}+\epsilon }$ is the simplest interpolation between the small and large *ϵ* limits. We demonstrate the accuracy of this simplified solution in Figures 2 and 3, where we compare the meridional velocity amplitude $\tilde{v}$ calculated using Matsuno's formula (red line, upper row), L‐H's formulae (purple lines, middle row), and the ad hoc formulae of the present sections (green lines, bottom row) for *ϵ* = 10^4^ and *ϵ* = 16, respectively. For comparison, the numerical meridional velocity amplitude calculated as explained in Section 2.2 is also shown (blue dots). In both figures, the comparison is made for a fixed value of *ϵ* and three values of *k*, corresponding to a small value of *k*/*ϵ* ^1/4^ = 2 (left column), a moderate value of *k*/*ϵ* ^1/4^ = 5 (middle column), and a large value of *k*/*ϵ* ^1/4^ = 8 (right column). At large *ϵ* (Figure 2), the ad hoc solution remains accurate for all values of *k* up to *k*/*ϵ* ^1/4^ = 8, whereas Matsuno's and L‐H's formulae are noticeably inaccurate even at moderate values of *k*/*ϵ* ^1/4^ = 5. On the other hand, at small *ϵ* (Figure 3), the ad hoc solution is inaccurate at small values of *k*/*ϵ* ^1/4^ = 2, whereas L‐H's formulae remains accurate for all *k* down to *k*/*ϵ* ^1/4^ = 2.

## 4 THE SPECIAL SOLUTION FOR ω = −K

As is the case of Matsuno (1966)'s solution on the *β*‐plane, the ad hoc solution obtained in Section 3 is only valid for *ω* ≠ −*k*. However, unlike the *β*‐plane, where *ω* = −*k* corresponds to infinite zonal velocity, on the sphere the zonal velocity is regular for all *k* and *ω*, suggesting that a solution with *ω* = −*k* may exist on the sphere.

**Figure 4:**
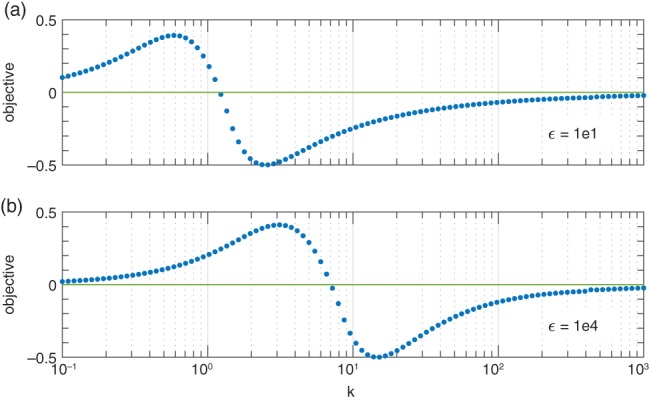
The objective function defined in Equation 5 obtained by integrating Equation 4 with ω = −k. The integration was performed as described in Section 2.2. Note that, since ω = −k, the objective is now a function of k. Results are shown for (a) ϵ = 10 and (b) ϵ = 10^4^

The existence of a solution with *ω* = −*k* is also evident in the numerical results shown in Figure 4, where the calculated objective function of Equation 5 obtained by solving Equation  4 with *ω* = −*k* is plotted over four decades of *k* (note that, since *ω* = −*k*, the objective function is now a function of *k*). Recall that the solutions of Equation 4 correspond to the roots of this objective function. Thus, the smooth shape of the calculated objective function in Figure 4 indeed substantiates the existence of a solution with *ω* = −*k* and further suggests that it is unique (for a given *ϵ*).

We now derive an analytic solution for Equation 4 with *ω* = −*k*, valid in the asymptotic limit *ϵ* ≫ 1. Differentiating the first row of Equation 4 with respect to *ϕ* and using the first and second rows to eliminate $\tilde{\eta }$ and $d\tilde{\eta }/d\phi$, respectively, yields the following second‐order equation for $\tilde{V}$:

(21) $$\begin{array}{ll} -\frac{d^{2}\tilde{V}}{d\phi^{2}}+ & \tan\phi \left(\frac{\Gamma_{+}(\phi )}{\Gamma_{-}(\phi )}\right)\frac{d\tilde{V}}{d\phi }+\left\{\sqrt{\epsilon }\frac{k}{\omega }-\omega^{2}+\frac{k^{2}}{\cos^{2}\phi }\right.\;\\ & \left.+\epsilon \sin^{2}\phi +\sqrt{\epsilon }\frac{k}{\omega }\tan^{2}\phi [1-(\frac{\Gamma_{+}(\phi )}{\Gamma_{-}(\phi )})]\right\}\tilde{V}=0, \end{array}$$

where

(22) $$\Gamma_{\pm }(\phi )=\frac{k^{2}\pm \omega^{2}\cos^{2}\phi }{\cos\phi }.$$

Rewriting Equation 21 in terms of the new independent variable x=sinϕ and substituting *ω* = −*k* yields

(23) $$-(1-x^{2})\frac{d^{2}\tilde{V}}{dx^{2}}+\frac{2}{x}\frac{d\tilde{V}}{dx}+\left\{\sqrt{\epsilon }+k^{2}\frac{x^{2}}{1-x^{2}}+\epsilon x^{2}\right\}\tilde{V}=0.$$

We seek solutions of the form

(24) $$\tilde{V}(x)=\exp\left[-\frac{\sqrt{b}x^{2}}{2}\right]f(\sqrt{b}x^{2}),$$

where the function *f* cannot increase faster than algebraically (so that $\tilde{V}$ is regular at the poles) and where

(25) $$\sqrt{b}=\frac{2(\epsilon +k^{2})}{1+\sqrt{1+4(\epsilon +k^{2})}},$$

which yields the following second‐order equation for the unknown function *f*:

(26) $$\begin{array}{ll} y & \frac{d^{2}f}{dy^{2}}-\left[\frac{1}{2}+y\right]\frac{df}{dy}=\frac{y^{2}}{\sqrt{b}}\frac{d^{2}f}{dy^{2}}+\left[\frac{y}{2\sqrt{b}}-\frac{y^{2}}{\sqrt{b}}\right]\frac{df}{dy}\;\\ & +\left[\frac{y^{2}}{4\sqrt{b}}\left(1+\frac{k^{2}}{b-y\sqrt{b}}\right)-\frac{c}{4\sqrt{b}}\right]f, \end{array}$$

where $y=\sqrt{b}x^{2}$ and $c=\sqrt{b}-\sqrt{\epsilon }$. Without loss of generality, we assume *f*(0) = 1, that is, $\max_{y}\{\tilde{V}\}=1$, as in the previous section.

The above steps are exact transformations of the original Equation 4 for the particular case when *ω* = −*k*, since these steps involve no approximations. In what follows, we consider *ϵ* ≫ 1 (which implies (see Equation (25)) that *b*≈*ϵ* and that *c* is O(1)) and seek approximate solutions in the form of a power series in $1/\sqrt{\epsilon }$, that is, we let

(27) $$f=f^{\left(0\right)}+\frac{1}{\sqrt{\epsilon }}f^{\left(1\right)}+\frac{1}{\epsilon }f^{\left(2\right)}+\text{⋯}$$

The resulting equation for the zeroth‐order term (which is just the left‐hand side of Equation (26)) is the confluent hypergeometric equation,

(28) $$y\frac{d^{2}f^{\left(0\right)}}{dy^{2}}-[\frac{1}{2}+y]\frac{df^{\left(0\right)}}{dy}=0.$$

The two independent solutions of this equation are *F*(0, − 1/2,*y*) = 1 and *y* ^3/2^ *F*(3/2,5/2,*y*), where *F*(*a*,*b*,*y*) is the confluent hypergeometric function (see e.g. (Abramowitz and Stegun, 1964)). The latter solution grows exponentially with *y* and is therefore rejected as a leading‐order solution of Equation 26. Therefore the only physically acceptable solution for the zeroth‐order approximation is *f* ^(0)^(*y*) = *const*. In accordance with the normalization chosen above, we let *f* ^(0)^(*y*) = 1, which implies that *f* ^(*i*)^(0) = 0 for all *i* > 0.

Substituting *f* ^(0)^ = 1 for the zeroth‐order approximation yields the following equation for the first‐order correction:

(29) $$y\frac{d^{2}f^{\left(1\right)}}{dy^{2}}-\left[\frac{1}{2}+y\right]\frac{df^{\left(1\right)}}{dy}=\left[\frac{y^{2}-c}{4}\right].$$

Dividing by *y* and defining *h*(*y*) = *df* ^(1)^/*dy* yields

(30) $$\frac{dh}{dy}=\left(\frac{1}{2y}+1\right)h+\frac{y^{2}-c}{4y}.$$

For the right‐hand side of this equation to be regular at *y* = 0, *h* has to satisfy *h*(0) = *c*/2 and the substitution of this expression for *h*(0) in the left‐hand side then yields *dh*(0)/*dy* = *c*. The general solution of this equation that satisfies *h*(0) = *c*/2 is

(31) $$h(y)=\frac{c}{2}+cy+\frac{(4c+1)\exp(y)\sqrt{y}}{4}\int_{0}^{y}\exp(-y^{\prime })\sqrt{y^{\prime }}\;dy^{\prime }.$$

The requirement that this solution does not grow exponentially with *y* yields *c* = −1/4. Integrating *h*(*y*) to obtain *f* ^(1)^(*y*) and using the condition *f* ^(1)^(0) = 0 (as explained above), it follows that the meridional structure to first order in $1/\sqrt{\epsilon }$ is

(32) $$\tilde{V}(y)\approx \exp\left[-\frac{y}{2}\right]\left(1-\frac{y}{8}-\frac{y^{2}}{8}\right).$$

Recall that $y\propto \sin^{2}\phi$, so that the above expression is meridionally symmetric about the equator. From the definition of *b* in Equation 25, we observe that

(33) $$1+\frac{1}{\sqrt{\epsilon }}-\frac{c}{\epsilon }-\frac{\epsilon +k^{2}}{\epsilon }\left(1-\frac{2c}{\sqrt{\epsilon }}\right)=0.$$

To leading order in *ϵ*, this equation has a solution for which *k* ^2^ is of order *ϵ* ^1/2^. The positive root, denoted by *k* ^⋆^, satisfies

(34) $$k^{⋆}=\frac{\epsilon^{1/4}}{\sqrt{2}}.$$

**Figure 5:**
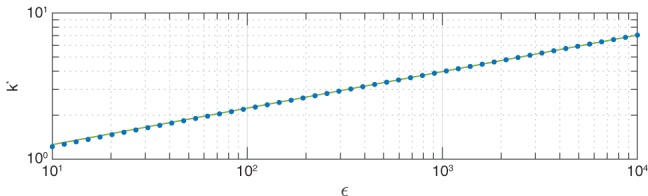
The location of the ω = −k solution as a function of ϵ. (Blue) dots: numerically calculated solution obtained by finding the single root of the objective function as described in Section 2.2. (Green) line: analytically calculated solution obtained using Equation 34

This result is confirmed numerically in Figure 5, where the location of the numerically calculated root of the objective function (blue dots) is compared with the analytically predicated location given by Equation 34 (green line). In addition, this value of *k* ^⋆^ reproduces exactly the value of *k* in Matsuno (1966)'s planar theory, where the inertia–gravity *n* = 0 mode changes to the Rossby *n* = 0 mode.

## 5 A COMPLEMENTARY PERSPECTIVE ON THE MRG WAVE MODE FROM A SCHRÖDINGER EQUATION FORM

Having established the existence of the ad hoc mode, including its regular crossing of the *ω* = −*k* curve, we now turn to the classification of this mode in the context of the eigenmodes (Rossby and inertia–gravity) on a sphere. Recently, the authors employed a Schrödinger equation form in the classification of eastward‐propagating waves on a sphere. In this section, we use the same Schrödinger equation approximation to provide a complementary perspective on the MRG wave by identifying it with the ground‐state solution of the corresponding Schrödinger equation. In particular, we obtain two Schrödinger equations, one for *ω* ^2^ ≪ *k* ^2^ and one for *k* = 0. By comparing the resulting analytic expressions with the numerical solutions, we show that the ground‐state solutions of these equations correspond to the MRG wave in their respective areas of validity.

Changing $\tilde{V}$ in Equation 21 to the new dependent variable *ψ*, defined by

(35) $$\tilde{V}=\Gamma_{-}^{1/2}(\phi )\psi ,$$

where Γ_−_ is defined in Equation 22, and using the relation $d\Gamma_{\pm }(\phi )/d\phi =\tan\phi \Gamma_{\mp }(\phi )$ yields the following “Schrödinger‐like” equation for *ψ*:

(36) $$\begin{array}{ll} -\frac{d^{2}\psi (\phi )}{d\phi^{2}} & +\left\{\epsilon \sin^{2}\phi +\frac{k^{2}}{\cos^{2}\phi }-\frac{1}{2}\frac{1}{\cos^{2}\phi }\left(\frac{\Gamma_{+}(\phi )}{\Gamma_{-}(\phi )}\right)\right.\;\\ & -\frac{1}{2}\tan^{2}\phi +\frac{3}{4}\tan^{2}\phi {\left(\frac{\Gamma_{+}(\phi )}{\Gamma_{-}(\phi )}\right)}^{2}\;\\ & +\left.\sqrt{\epsilon }\frac{k}{\omega }\tan^{2}\phi \left[1-\left(\frac{\Gamma_{+}(\phi )}{\Gamma_{-}(\phi )}\right)\right]\right\}\psi (\phi )\;\\ & =\left\{\omega^{2}-\sqrt{\epsilon }\frac{k}{\omega }\right\}\psi (\phi ).\; \end{array}$$

We refer to this equation as a “Schrödinger‐like” equation, since the unknown frequency *ω* appears in both the *ϕ*‐dependent terms on the left‐hand side and the constant terms on the right‐hand side. A proper Schrödinger equation is obtained from Equation 36 in the following two cases.

1. For *k* ≥ 1 and *ω* ^2^ ≪ *k* ^2^, which corresponds to slow Rossby waves, $\omega^{2}\cos^{2}\phi$ can be uniformly neglected compared with *k* ^2^, so according to Equation 22Γ_+_/Γ_−_→ + 1, which yields the following (time‐independent) Schrödinger equation for *ψ*:
  (37) $$\left[\;-\frac{d^{2}}{d\phi^{2}}+\frac{k^{2}-1/4}{\cos^{2}\phi }+\epsilon \sin^{2}\phi \;\right]\psi =\left[\omega^{2}-\sqrt{\epsilon }\frac{k}{\omega }+\frac{1}{4}\right]\psi .$$
2. For *k* = 0 and *ω* ≠ 0, which corresponds to zonally symmetric waves, Γ_+_/Γ_−_ = −1, which yields the following (time‐independent) Schrödinger equation for *ψ*:
  (38) $$\left[\;-\frac{d^{2}}{d\phi^{2}}+\frac{3/4}{\cos^{2}\phi }+\epsilon \sin^{2}\phi \;\right]\psi =\left[\omega^{2}+\frac{1}{4}\right]\psi .$$

Observe that the Hamiltonian on the left‐hand side of Equation 38 is obtained from the one on the left‐hand side of Equation 37 by setting *k* = 1. Thus, it suffices to study the following “generic” Schrödinger equation:

(39) $$\left[\;-\frac{d^{2}}{d\phi^{2}}+\frac{k^{2}-1/4}{\cos^{2}\phi }+\epsilon \sin^{2}\phi \;\right]\psi =E\psi .$$

Once the eigenfunctions and corresponding energies of this generic Schrödinger equation are found, the eigensolutions of the zonally symmetric waves are obtained by setting *k* = 1 in the eigenfunctions and *k* = 0 in the energies. Once the energies are obtained, the frequencies of the Rossby waves are obtained from the cubic relations,

(40) $$\omega^{3}+\left(\frac{1}{4}-E\right)\omega -\sqrt{\epsilon }k=0,$$

and the frequencies of the zonally symmetric waves are obtained by setting *k* = 1 in the energies and using the quadratic relations:

(41) $$\omega^{2}+\left(\frac{1}{4}-E\right)=0.$$

The solution of the generic Schrödinger Equation 39 (along with the associated boundary conditions of vanishing *ψ* at the poles) is composed of a countable set of eigenfunctions *ψ*_*n*_ and corresponding energies *E*_*n*_, where *n* = 0,1,2,…. Thus, in addition to the zonal wavenumber *k*, each wave mode is also characterized by a mode number *n* that enumerates the eigensolutions of the Schrödinger equation. Since the meridional structure of the wave amplitudes (given by the eigenfunctions) is not periodic, *n* is not a wavenumber and is therefore referred to in this work as the meridional mode number. As in all Sturm–Liouville problems, the number of (internal) zero‐crossings (that is, sign changes) of the eigenfunction *ψ*_*n*_ is equal to the mode number *n*. Since, in both cases considered here, Γ_−_ has no (internal) zero‐crossings, the meridional velocity $\tilde{V}$ has the same number of zero‐crossings as *ψ*. This fact suggests that the ad hoc solution should be classified as the *n* = 0 mode of the Schrödinger equation, since $\tilde{V}$ has no zero‐crossings.

As discussed in De‐Leon and Paldor (2011), the low energy‐states of the generic Schrödinger equation correspond to equatorially trapped waves, the latitude‐dependent amplitudes of which become negligible outside a narrow equatorial band. In particular, for *n* = 0 we consider the leading (second) order terms in the expansion of the potential on the left‐hand side of the generic Schrödinger equation in a power series in *ϕ* around the equator. The resulting equation is

(42) $$-\frac{d^{2}\psi }{d\phi^{2}}+\left[\;\left(k^{2}-\frac{1}{4}+\epsilon \right)\phi^{2}\;\right]\psi =\left[\;E(\omega )-k^{2}+\frac{1}{4}\;\right]\psi ,$$

which is analogous to the well‐known Schrödinger equation of the quantum harmonic oscillator with Hamiltonian

(43) $$-\frac{\hbar^{2}}{2m}\frac{d^{2}}{dx^{2}}+\frac{1}{2}m{\tilde{\omega }}^{2}x^{2},$$

where *ℏ* is Planck's constant and *m* and $\tilde{\omega }$ are the effective mass and angular frequency of the system, respectively. Using the solutions of Equation 43, which are readily found in any text book on quantum mechanics (e.g. Sakurai and Napolitano 2011), and carrying out the required adjustments yields the following energies and corresponding eigenfunctions:

(44a) $$\begin{array}{ll} E_{n}(\omega ) & =\sigma^{2}(2n+1)+k^{2}-\frac{1}{4},n=0,1,2,\text{⋯}, \end{array}$$

(44b) $$\begin{array}{ll} \psi_{n}(\phi ) & =\exp\left[-\frac{{\left(\sigma \phi \right)}^{2}}{2}\right]{\tilde{H}}_{n}\left(\sigma \phi \right), \end{array}$$

where *σ* = (*k* ^2^ − 1/4 + *ϵ*)^1/4^ and ${\tilde{H}}_{n}$ are the normalized Hermite polynomials of degree *n* (Abramowitz and Stegun, 1964).

**Figure 6:**
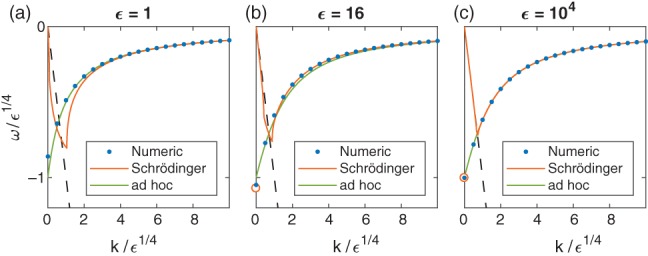
A comparison between the numerically computed (blue dots) dispersion relation ω(k) of the MRG wave mode, the analytic solution obtained from the Schrödinger Equation 44 (orange line), and the ad hoc solution obtained in Section 3 (green line). The dispersion relation used for the ad hoc solution in this figure is taken from Equations 20 and 16 for ϵ = 1 in panel (a) and ϵ = 10^4^ in panel (c), respectively. For ϵ = 16, panel (b), the expression in Equation 16 is used, since it is the more accurate of the two. The numeric values were obtained using the shooting method, as explained in Section 2.2. The (black) dashed line corresponds to ω = −k. The approximate analytic value at the ordinate (i.e. k = 0) in panel (a) falls occurs at −1.35 which is below the abscissa

The dispersion relation for the ad hoc solution obtained from Equation 44a by setting *n* = 0 and solving the cubic equation for *ω* ^2^ ≪ *k* ^2^ (orange line) or the quadratic equation for *k* = 0 (orange open circle) is compared with the numerical solution (blue dots) for *ϵ* = 1 (left panel), *ϵ* = 16 (middle panel) and *ϵ* = 10^4^ (right panel) in Figure 6. Tracing down the dispersion curve from large values of *k*, it can be seen that the ground‐state solution of Equation (37) fits the numeric and ad hoc solutions all the way to the vicinity of the *ω* = −*k* line, which suggests that the approximated Schrödinger equation can be used to describe the numeric and ad hoc solutions for *ω* ^2^ < *k* ^2^, and not just *ω* ^2^ ≪ *k* ^2^ as was originally stipulated. On the other hand, in the vicinity of the *ω* = −*k* line, the ground‐state solution curves abruptly upwards towards the origin along a curve that nearly parallels the *ω* = −*k* line, which, as shown in Section 4, is not a solution of Equation 4, except for a single point at $\omega =-k=\epsilon^{1/4}/\sqrt{2}$. Along the *k* = 0 ordinate, the MRG wave mode fits the ground‐state solution of Equation 38 (again with higher accuracy for large *ϵ*). Thus, the ad hoc solution connects smoothly between the *n* = 0 Rossby‐wave mode and the *n* = 0 zonally symmetric (that is, *k* = 0) WIG mode, both of which arise naturally from the Schrödinger equation formulations. Therefore, the ad hoc solution should be classified as the mixed Rossby–gravity wave.

## 6 SUMMARY

The analytic and numerical results presented in the preceding sections clearly demonstrate that the MRG mode, developed straightforwardly on the equatorial *β*‐plane in Matsuno (1966), also exists on the sphere. However, even though the equatorial *β*‐plane approximates the sphere in the *ϵ*→*∞* limit, the planar dispersion relation approximates that accurately on a sphere even at *ϵ* = 16. In contrast, the planar eigenfunctions approximate the spherical eigenfunctions only for *ϵ* = 10^4^.

Of particular relevance to the present study is the comprehensive spherical theory of Longuet‐Higgins (1968), where the MRG mode appears in the numerical calculations (see panels b in Figures 2, 3, 4, 5, 6) but is not designated as a particular mode in the description of the modes given in the text. As we show in Figure 2, L‐H's *ϵ*→*∞* solution works well only for small *k*, whereas his *ϵ*→0 solution works well for most *k* (see the middle panels in Figure 3).

Our results show that the meridional velocity of the MRG mode on the sphere is well‐described by $\cos^{\gamma }(\phi )$, where *γ* is given by Equation 15 (along with Equation (12)). As is evident from Equation 15, *γ* is approximated by $\sqrt{k^{2}+\epsilon }$, which was also noted by Boyd (2018). In fact, the ad hoc solution fits the numerical solutions for nearly all values of *ϵ* and *k*, except when *ϵ* and *k* are both O(1) (recall that on a sphere only integer values of *k* are physically acceptable). The global applicability of our $\cos^{\gamma }(\phi )$ solution at both small and large *ϵ* clarifies the nature of the mixed mode. For not‐too‐fast rotation rate (that is, small *ϵ*), the mixed mode is a pure Rossby wave for nearly all *k* (see left panel of Figure 6). If we increase the rotation rate adiabatically, then the mode gradually becomes a non‐Rossby mode over an increasing range of *k* values. Thus, at sufficiently low *k* (the specific *k* value depends on *ϵ*) the (inertia–gravity segment of the) mixed mode can be viewed as a long Rossby wave distorted by the fast rotation and gravity. This conclusion is implicit in L‐H's numerical solutions, but the present work is the first to provide a theory for this change in character of the mixed mode with the increase in *ϵ* at a fixed value of *k*.

The new feature of the MRG mode on a sphere is its regular crossing of the *ω* = −*k* curve (at $k=\epsilon^{1/4}/\sqrt{2}$). In contrast, on the equatorial *β*‐plane the zonally propagating wave solution is associated with singular zonal velocity at *ω* = −*k* (more generally at *ω* ^2^ = *k* ^2^).

The main assumptions and results of the present study are summarized in Table 1, where they are also compared with the main assumptions and results of Matsuno (1966) and Longuet‐Higgins (1968). Note that the meridional structure in Matsuno's theory is independent of *ϵ*. Also, the meridional structure of Longuet‐Higgins' theory is independent of *ϵ* at small *ϵ* and independent of *k* at large *ϵ*. In contrast, in the present theory the dependence of *γ* on *k* and *ϵ* is highly nonlinear.

**Table 1:** A summary of the assumptions and results of the present study compared with those of Matsuno (1966) and Longuet‐Higgins (1968)

		**Longuet‐Higgins (** 1968 **)**		**Present work (ad hoc)**	
	**Matsuno 1966**	***ϵ*→0**	***ϵ*→*∞***	***ϵ*→0**	***ϵ*→*∞***
Assumptions	*β*‐plane	Sphere		Sphere	
Limitations	*ω* ≠ −*k*	–	k=O(1)	–	–
Frequencies	$\frac{k}{2}-\sqrt{{\left(\frac{k}{2}\right)}^{2}+\sqrt{\epsilon }}$	$-\frac{\sqrt{\epsilon }}{k+1}$	*k* − *ϵ* ^1/4^	$-\frac{\sqrt{\epsilon }}{k+1}$	$\frac{k}{2}-\sqrt{{\left(\frac{k}{2}\right)}^{2}+\sqrt{\epsilon }}$
Meridional structure	$e^{\left(-\epsilon^{1/2}y^{2}/2\right)}$	$\cos^{k}\phi$	$e^{\left(-\epsilon^{1/2}\sin^{2}\phi /2\right)}$	$\cos^{\gamma }\phi$	$\cos^{\gamma }\phi$

## ACKNOWLEDGEMENT

Financial support for this work was provided by ISF grant No. 1558/14 and by a European Research Council starting grant under the European Union's Horizon 2020 research and innovation programme (grant agreement No. 677756) to HU (CG).
